# When Cortical Bone Matrix Properties Are Indiscernible between Elderly Men with and without Type 2 Diabetes, Fracture Resistance Follows Suit

**DOI:** 10.1002/jbm4.10839

**Published:** 2023-12-01

**Authors:** Eva M. Wölfel, Benjamin Bartsch, Jasmin Koldehoff, Imke A. K. Fiedler, Sofie Dragoun‐Kolibova, Felix N. Schmidt, Johannes Krug, Mei‐Chun Lin, Klaus Püschel, Benjamin Ondruschka, Elizabeth A. Zimmermann, Hans Jelitto, Gerold Schneider, Bernd Gludovatz, Björn Busse

**Affiliations:** ^1^ Department of Osteology and Biomechanics University Medical Center Hamburg‐Eppendorf Hamburg Germany; ^2^ Institute of Advanced Ceramics Hamburg University of Technology Hamburg Germany; ^3^ Interdisciplinary Competence Center for Interface Research (ICCIR) University Medical Center Hamburg‐Eppendorf Hamburg Germany; ^4^ Institute of Legal Medicine University Medical Center Hamburg‐Eppendorf Hamburg Germany; ^5^ Faculty of Dental Medicine and Oral Health Sciences McGill University Montreal Canada; ^6^ School of Mechanical and Manufacturing Engineering University of New South Wales, Sydney (UNSW Sydney) Sydney Australia

**Keywords:** BONE MATERIAL QUALITY, CORTICAL BONE, FRACTURE TOUGHNESS, MECHANICAL TESTING, T2DM

## Abstract

Type 2 diabetes mellitus (T2DM) is a metabolic disease affecting bone tissue and leading to increased fracture risk in men and women, independent of bone mineral density (BMD). Thus, bone material quality (i.e., properties that contribute to bone toughness but are not attributed to bone mass or quantity) is suggested to contribute to higher fracture risk in diabetic patients and has been shown to be altered. Fracture toughness properties are assumed to decline with aging and age‐related disease, while toughness of human T2DM bone is mostly determined from compression testing of trabecular bone. In this case‐control study, we determined fracture resistance in T2DM cortical bone tissue from male individuals in combination with a multiscale approach to assess bone material quality indices. All cortical bone samples stem from male nonosteoporotic individuals and show no significant differences in microstructure in both groups, control and T2DM. Bone material quality analyses reveal that both control and T2DM groups exhibit no significant differences in bone matrix composition assessed with Raman spectroscopy, in BMD distribution determined with quantitative back‐scattered electron imaging, and in nanoscale local biomechanical properties assessed via nanoindentation. Finally, notched three‐point bending tests revealed that the fracture resistance (measured from the total, elastic, and plastic J‐integral) does not significantly differ in T2DM and control group, when both groups exhibit no significant differences in bone microstructure and material quality. This supports recent studies suggesting that not all T2DM patients are affected by a higher fracture risk but that individual risk profiles contribute to fracture susceptibility, which should spur further research on improving bone material quality assessment in vivo and identifying risk factors that increase bone fragility in T2DM. © 2023 The Authors. *JBMR Plus* published by Wiley Periodicals LLC on behalf of American Society for Bone and Mineral Research.

## Graphical abstract

Bone quality assessment, along with fracture resistance measurements, was conducted in cortical bone samples from male individuals with and without type 2 diabetes mellitus (T2DM). The analysis of bone material quality revealed that both the control and T2DM groups exhibited no significant differences, which is also reflected in the indiscernible fracture resistance. These data support recent studies suggesting that not all T2DM patients but rather subgroups of patients experience an increased fracture risk potentially due to a specific risk profile

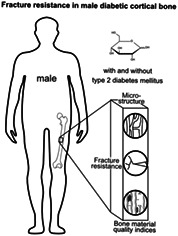

## Introduction

Type 2 diabetes mellitus (T2DM), a metabolic disease with increasing prevalence expected to affect an estimated 578 million adults worldwide by 2030, leads to various complications, including bone fragility, indicated by a relative risk of 1.2 for hip and 1.17 for any fracture.^(^ ^1^ ^)^ The increased fracture risk is not directly linked to bone mineral density (BMD), so BMD fails to predict diabetic patients at increased risk of fracture.^(^ ^2^ ^)^ Currently, clinical in vivo bone diagnostic tools, such as dual energy X‐ray absorptiometry (DXA), high‐resolution peripheral quantitative computed tomography (HR‐pQCT), and impact indentation, are not sufficiently sensitive to identify and predict T2DM patients at increased fracture risk, whereas impaired bone material quality, i.e., bone material properties affecting bone toughness without attribution to bone mass or quantity, has been proposed to contribute to higher fracture risk in T2DM.^(^ ^3^ ^)^

A higher cortical porosity subgroup measured with micro–CT (μCT) was identified ex vivo and associated with higher mineralization heterogeneity in human femoral cortical bone,^(^ ^4^ ^)^ as well as higher lab‐based cyclic indentation depth but unchanged in vivo impact indentation properties in the tibia.^(^ ^5^ ^)^ A higher cortical porosity subgroup measured with HR‐pQCT in vivo was also associated with fragility fractures.^(^ ^6^, ^7^ ^)^ Independent of microstructural differences, higher accumulation of advanced glycation end products (AGEs), such as carboxymethyl‐lysine, and lower collagen fibril deformation were measured in human T2DM cortical bone compared to nondiabetic bone.^(^ ^4^, ^8^ ^)^ Higher cyclic indentation depth was also observed in femoral neck cortical bone from T2DM patients, with a trend toward higher AGE accumulation but no differences in compressive biomechanical properties in trabecular bone.^(^ ^9^ ^)^ Studies on trabecular bone cores from the femoral head of T2DM patients showed lower bone volume and trabecular thickness, lower mineral content, and lower mineral‐to‐matrix ratio in combination with lower toughness measured with compression testing,^(^ ^10^ ^)^ while others found higher pentosidine content and higher mineral‐to‐matrix ratio, in combination with a higher modulus but no difference in toughness, determined using compression testing of trabecular bone from T2DM male patients.^(^ ^11^ ^)^ Individual tissue‐level changes at different length scales, such as microstructural changes or changes in bone matrix composition, contribute to bone overall fracture susceptibility. The aforementioned studies are indicative for altered bone material quality in patients with T2DM that can partly be linked to changes in toughness at the tissue level in trabecular bone. However, data on the fracture behavior in human diabetic cortical bone tissue are currently limited.

Three‐point bending tests of notched bone samples enable characterization of the fracture properties of bone tissue and allow for the analysis of bone resistance to the initiation and propagation of cracks. Furthermore, the combination of such testing with imaging techniques, such as optical or electron microscopy, enables the visualization of crack growth and the assessment of relevant bone toughening mechanisms.^(^ ^12^ ^)^ However, this requires relatively large sample dimensions, which is often a limitation of bone research. Furthermore, different three‐point bending setups, such as under vacuum using a scanning electron microscope for crack visualization or in a normal environment using an optical microscope along with different sample sizes and testing properties, have been applied, making comparisons across studies difficult.^(^ ^13^ ^)^ While standardizations of three‐point bending of nonbiological materials exist and are documented, for example, in ASTM International's Standard Test Method for Measurement of Fracture Toughness E 1820, no standardization for human bone as biomaterial is available across laboratories or countries. Nevertheless, using three‐point bending tests, a significant reduction in fracture toughness in human cortical bone with age compared to young and middle‐aged individuals has been shown,^(^ ^14^, ^15^ ^)^ suggesting changes in fracture toughness in aging and possibly age‐related diseases such as diabetes mellitus. All fracture toughness properties measured in cadaveric human cortical bone were shown to decrease with age, although age explains only 13% to 23% of the variance, while other parameters, such as bound and pore water, as well as indentation resistance properties, best explain fracture toughness variance, suggesting a multimodal assessment of fracture toughness in bone.^(^ ^16^ ^)^ Using the same heterogeneous sample cohort, including three individuals with diabetes mellitus, two with osteoporosis, and six with cancer, a strong relationship was found between fracture toughness and collagen network connectivity.^(^ ^17^ ^)^ While these studies include a large sample set (62 donors in total), only three donors were diabetic, and thus no firm conclusion can be drawn on diabetes‐induced impacts on fracture toughness.

Differences in bone material quality in T2DM have been shown for cortical and trabecular bone, and they have been linked to partially altered mechanical properties in trabecular bone. Yet reports on cortical bone toughness in T2DM are lacking in the literature. In this case‐controlled study, we therefore aimed to determine fracture toughness in T2DM cortical bone tissue in combination with a multiscale approach to assess the microstructure and bone material quality indices of the same bone tissue.

## Materials and Methods

### Study design

**Fig. 1:**
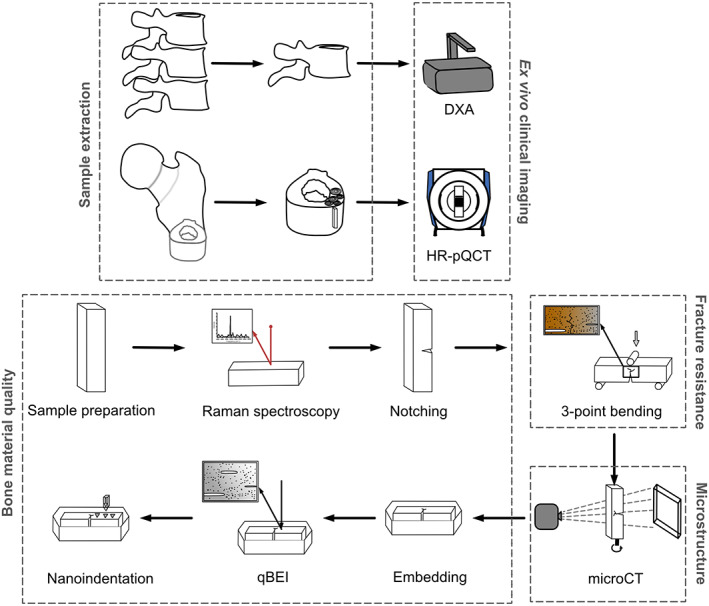
Study design. The 12th thoracic vertebra with adjacent vertebrae and the mid‐diaphysis of the femur were extracted from T2DM individuals and age‐matched healthy controls (sample extraction). The 12th thoracic vertebra was scanned using DXA, and the femoral cross‐sections were scanned with HR‐pQCT to apply ex vivo clinical imaging. Bending beam samples were prepared and scanned with Raman spectroscopy to assess bone material quality indices in terms of bone matrix composition. Samples were notched and fracture resistance was assessed using three‐point bending tests. Microstructure was analyzed using micro–CT, while further bone material quality indices were determined using quantitative back‐scattered electron microscopy and nanoindentation on embedded bone samples.

Femoral samples (*n* = 29) from the mid‐diaphysis as well as the 12th thoracic and adjacent vertebrae were obtained from male individuals during autopsy (Ethical Approval WT037/15). Approximately 1.5‐cm‐thick femoral cross‐sections (Fig. 1) were extracted from 18 healthy individuals (74.22 ± 6.67 years [range 61 to 89 years]) and 11 individuals diagnosed with T2DM (76.18 ± 6.62 years [range 69 to 89 years]). Following extraction, all samples were wrapped in phosphate‐buffered saline (PBS)‐soaked gauze and stored at −20°C until further analysis.

In both groups, inclusion criteria such as the absence of any bone‐related diseases, e.g., Paget's disease of bone, osteogenesis imperfecta, fibrous dysplasia or malignancy, were applied. Additionally, individuals with renal or hepatic disorders, hyperparathyroidism, as well as bedrest, and those with bone metabolism affecting medications such as thiazolidinediones, glucocorticoids, or bisphosphonates were strictly excluded. De‐identified data of all individuals were collected and included age, sex, weight, height, diabetes mellitus status, and, if available, antidiabetic treatment, which ranged from oral antidiabetics such as metformin to insulin treatment. Diabetes mellitus status was based on available medical and autopsy records but was limited to the presence of T2DM and, in some cases, diabetes medication.

### Dual energy X‐ray absorptiometry

Following an ex vivo protocol,^(^ ^18^ ^)^ the 12th thoracic vertebra was scanned with DXA (GE Healthcare, GER) to determine the osteoporotic status based on vertebral areal (aBMD) (Fig. 1). Compensation for missing soft tissue due to dissection was done by a water phantom provided by the manufacturer producing a water column of 15 cm simulating the thickness of soft tissue of a person with a body weight of 70 kg and height of 170 cm. The vertebrae were positioned in a plastic container filled with water, and the water phantom was placed on top. The values of aBMD for anterior–posterior (AP) and lateral (LAT) scan directions were obtained using the manufacturer's software. In one control sample and one T2DM sample, no 12th thoracic vertebra was available for logistical reasons.

### High‐resolution peripheral quantitative computed tomography

The extracted, frozen, mid‐diaphyseal femur samples were scanned in a HR‐pQCT system (Xtreme CT I, Scanco, Medical AG, Switzerland) following the removal of soft tissue (Fig. 1). Weekly and monthly calibration was performed using the manufacturer's calibration phantoms for quality control. Per sample, 12 mm of the mid region were scanned to avoid inclusion of regions with extraction artifacts. The scan was performed with 750 projections, a voxel size of 82 μm, and an integration time of 100 ms at each angular position over 180°. Following reconstruction, images were analyzed with the software provided by the manufacturer according to protocols previously published by Burghardt and colleagues^(^ ^19^ ^)^ and as described in our previous publication.^(^ ^5^ ^)^ The following cortical parameters were evaluated: cortical volumetric bone mineral density (Ct.vBMD, mg HA/cm^3^), cortical porosity (Ct.Po, %), cortical thickness (Ct.Th, mm), cortical pore diameter (Ct.Po.Dm, mm), cortical perimeter (Ct.Pm, mm), cortical area (Ct.Ar, mm^2^), and total area (Tt.Ar, mm^2^). However, three samples in the control group were incomplete and could therefore not be analyzed using HR‐pQCT.

### Sample preparation

The frozen femoral mid‐diaphyseal samples were sawed with a diamond band saw (EXAKT Technologies, Inc., USA) to extract smaller, rectangular bone samples of at least 12 mm in length from the lateral region of the femoral quadrant (Fig. 1). The smaller bone samples were further sawed using a low‐speed saw (Buehler Ltd., Germany) with a 300‐μm‐thick diamond blade to obtain bending beams of 2 mm thickness (*B*) × 3 mm height (*W*) × 12 mm length (*L*). The bone samples were then polished using 1‐μm diamond suspension to provide a final surface finish for imaging of the crack propagation during three‐point bending testing on one surface of the bending beam dedicated to crack imaging. All procedures were performed while keeping the bone samples hydrated in PBS.

### Raman spectroscopy

Raman micro‐spectroscopy was performed on moist and unfixed bone specimens (inVia, Renishaw, United Kingdom; Fig. 1). Samples were positioned on a microscope slide, and a 63× immersion objective was used to allow spectral acquisition while keeping samples hydrated in PBS, as previously performed,^(^ ^20^ ^)^ and to determine the region of interest in the middle of the bone sample. For each sample, spectral maps of 200 × 30 μm were acquired at a 5‐μm step size (287 spectra per sample) using the streamline mode with an integration time of 8 ms, a grating of 1200 lines per mm, a 785‐nm laser wavelength, and a spectral range of ~800 to 1800 cm^−1^. During the whole scan time, the bone samples were kept humid in PBS. Spectra processing was done using the WiRE software provided by the manufacturer and a custom‐made MATLAB code (MATLAB, R2019b, MathWorks Inc., USA) and included removal of cosmic rays, averaging of all spectra per sample, and linear baseline removal under the peaks of interest. The following previously established parameters were obtained^(^ ^21^, ^22^, ^23^ ^)^: mineral‐to‐matrix ratios (phosphate ν_1_ peak at 962 cm^−1^/amide I peak envelope between 1600 and 1720 cm^−1^; phosphate ν_1_ peak at 962 cm^−1^/amide III envelope between 1215 and 1365 cm^−1^), carbonate‐to‐phosphate ratio (carbonate peak at 1070 cm^−1^/ν_1_ phosphate peak), carbonate‐to‐amide I peak (carbonate peak/amide I envelope), crystallinity calculated with the reciprocal of the full width at half maximum (FWHM) of the ν_1_ phosphate peak, and amide I subpeak ratio 1666/1685 cm^−1^ based on the fitting of amide I subpeaks.

### Sample notching

Following Raman spectroscopy, the cortical bone tissue was notched according to previously described protocols^(^ ^14^ ^)^ (Fig. 1). In brief, the first part of the notch was induced using a low‐speed saw with 300‐μm blade thickness. A razor blade was inserted into the notch and the final notch length of approximately half the sample height *W* was prepared with 1‐μm diamond suspension. Using an optical microscope, the notch length for every sample was measured and recorded on the surface selected for crack imaging.

### Three‐point bending

The prepared bone samples were incubated in PBS overnight to avoid an additional freeze–thaw cycle and sufficiently hydrate the bone tissue. Three‐point bending tests (Fig. 1) were performed at room temperature in ambient air using a custom‐made three‐point bending machine^(^ ^24^ ^)^ with 10 mm support span and round contact points of 1‐mm radius. During testing, images of the bone surface were taken using a 400× optical microscope objective to record crack propagation. Furthermore, the samples were continuously moistened with PBS. The samples were preloaded to 2 N at a displacement rate of 2 μm/s. Testing was conducted at an initial displacement rate of 1 μm/s to a load of 15 N. Subsequently, the displacement rate was reduced to 0.15 μm/s. During the entire measurement, force and displacement were continuously recorded using a custom‐made software interface in LabView collecting one data point per second. To enable stable crack growth, the testing machine used an automatic control system, which sensed the beginning of crack growth by a decrease in the slope of the recorded force–displacement curve; a slope of −0.45 N/μm over the last 15 data points resulted in a partial unloading of the sample followed by manual restart of the loading. Each test was terminated after a crack length of approximately 700 μm (i.e., ~47% of the remaining ligament *b*) was observed.

### Data analysis

The recorded images were analyzed using ImageJ to determine the crack length for each increment of crack growth. Load–displacement curves were exported from LabView. Determination of the area under the load–displacement curve, curve fitting, and determination of the 95% confidence interval were performed using a custom‐made MATLAB script (MATLAB, R2023a, MathWorks Inc., USA).

Bone shows not only elastic but also plastic deformation during mechanical loading. To account for both elastic and plastic deformation, the nonlinear elastic J‐integral was measured. The total J‐integral was computed as the sum of elastic and plastic components. In this way the J‐integral at any point of the load–displacement curve can be determined according to ASTM E1820‐01. The elastic J‐integral (*J*_*el*_, J/m^2^) is calculated as

$$J_{\mathrm{el}}=\frac{K_{I}^{2}\left(1-\nu^{2}\right)}{E}.$$

with the stress intensity factor (*K*_*I*_) calculated according to ASTM E1820‐01 using a Poisson ratio of ν = 0.3^(^ ^25^ ^)^ and an E‐modulus of E = 19.5 GPa, based on our nanoindentation results.

$$K_{I}=\frac{\mathrm{PS}}{BW^{\frac{3}{2}}}\;f\left(\frac{a}{W}\right),$$

K_I_ is calculated based on the measured load *P*, the support span *S* = 10 mm, the sample thickness *B*, the sample height *W*, and the geometric factor $f\left(\frac{a}{W}\right)$ according to ASTM E1820‐01.

$$f\left(\frac{a}{W}\right)=\frac{3{\left(\frac{a}{W}\right)}^{\frac{1}{2}}[1.99-\left(\frac{a}{W}\right)\left(1-\frac{a}{W}\right)\times (2.15-3.93\left(\frac{a}{W}\right)+2.7\left(\frac{a^{2}}{W^{2}}\right)]}{2\left(1+2\frac{a}{W}\right){\left(1-\frac{a}{W}\right)}^{\frac{3}{2}}}.$$

The plastic J‐integral (*J*_*pl*_, J/m^2^) was calculated by dividing the plastic area underneath the load–displacement curve by the uncracked ligament width multiplied by the width of the sample based on the following formula:

$$J_{\mathrm{pl}}=\frac{2A_{\mathrm{pl}}}{B\left(W-a\right)},$$

with *A*_*pl*_ being the area under the load–displacement curve between two crack growth events enclosed by parallel lines with an identical slope to the loading slope prior to the first crack extension.

Finally, the total J‐integral (*J*_*tot*_, J/m^2^) was determined:

$$J_{\mathrm{tot}}=J_{\mathrm{el}}+J_{\mathrm{pl}}.$$

### Micro–CT

Directly after fracture toughness testing, the cortical bone samples were fixed in 4% buffered paraformaldehyde for 1 week followed by storage in PBS. To determine cortical porosity (Ct.Po, %) and tissue mineral density (TMD, mg HA/cm^3^), the bone samples were scanned using a desktop μCT system (μCT 40, Scanco) using 6 μm resolution, 300 ms integration time, 55 kV X‐ray tube voltage, and 145 mA X‐ray tube current (Fig. 1). The image data were analyzed, and three‐dimensional (3D) renderings were obtained using Xamflow (version 1.8.09, Lucid Concepts AG, Switzerland). Additionally, canal parameters such as canal diameter and canal separation were evaluated based on the sphere fitting method.^(^ ^26^ ^)^

### Quantitative back‐scattered electron imaging

Following μCT imaging, the cortical bone samples were embedded in polymethyl methacrylate, grinded to a coplanar state, and polished to provide a smooth surface of the bone tissue. Quantitative back‐scattered electron imaging (qBEI) was performed using a scanning electron microscope (Zeiss crossbeam 340, Carl Zeiss AG, Germany) with a back‐scattered electron detector (Fig. 1). Prior to scanning, the embedded bone samples were sputtered with carbon. Gray scale images were obtained through a constant beam current determined using a Faraday cup and calibration standard with aluminum and carbon (MAC Consultants Ltd., England), in combination with a constant working distance of 20 mm and a voltage of 20 kV. In total, five images per sample around the crack were acquired and analyzed using a custom‐made MATLAB script routine (MATLAB, R2019b, MathWorks Inc., USA). Based on the BMD distribution the following parameters were determined: average calcium concentration (CaMean, wt%), most frequent calcium concentration (CaPeak, wt%), and mineralization heterogeneity, which is based on the standard deviation of the BMDdistribution curve (CaWidth, wt%).

### Nanoindentation

To determine the material properties of the bone tissue, nanoindentation was performed close to the crack surface (Fig. 1). The embedded bone sample surface was further polished using 3‐μm and 1‐μm diamond suspension and 0.5‐μm aluminum oxide suspension, followed by ultrasonic cleaning in distilled water. Using a Berkovich diamond tip in an iMicro nanoindenter (KLA instruments, CA, USA), 30 indentations in close vicinity to the crack path were obtained using depth‐sensing continuous stiffness mode and a final depth of 2000 nm. Prior to and following each measurement calibration of the tip was performed on fused silica. Based on the method by Oliver and Pharr^(^ ^27^ ^)^ and by applying a Poison's ratio of 0.3, hardness and Young's modulus were determined for eight control and five T2DM samples.

### Statistical analysis

GraphPad Prism (version 9, GraphPad Software, LLC, USA) was used for statistical analysis. The normal distribution of the data was verified using a Kolmogorov–Smirnov test. Normally distributed data were tested using a Student's *t* test, while nonnormally distributed data were analyzed using a Mann–Whitney U test. An alpha level below 0.05 was regarded as statistically significant.

## Results

Using a multiscale approach, we determined bone material quality indices in combination with fracture toughness in cortical bone samples from male individuals with T2DM (*n* = 11) and healthy age‐matched controls (*n* = 18). All individuals were male, ruling out any sex‐related differences. Sample characteristics are described in Table 1. Both groups did not differ significantly in age, BMI, and aBMD of the 12th thoracic vertebra measured with DXA assuring an absence of osteoporosis.

**Table 1:** Sample Characteristics of 18 Control and 11 T2DM Cases

	Control	T2DM
Number of samples	18	11
Age (years)	74.22 ± 6.67	76.18 ± 6.62
BMI (kg/m^2^)	25.63 ± 2.84	25.81 ± 3.3
Ex vivo aBMD of 12th	AP: 0.908 ± 0.279	AP: 0.856 ± 0.163
thoracic vertebra (g/cm^2^)	LAT: 0.681 ± 0.225	LAT: 0.657 ± 0.125
Diabetes medication		
Oral antidiabetics	‐	5 (45.5%)
Insulin	‐	1 (9%)
No information available	‐	5 (45.5%)

Neither group showed significant differences in age and BMI. Areal BMD was not significantly different between the groups in both scanning directions.

AP = anterior posterior; LAT = lateral.

Additionally, cortical bone microstructure was assessed as in a clinical setup using HR‐pQCT showing no significant differences in microstructure determined at a resolution of 82 μm; specifically, cortical vBMD was 1063 ± 43.58 and 1048 ± 27.7 mg HA/cm^3^, while cortical porosity was 2 ± 1.6 and 2 ± 1% for the control and T2DM groups, respectively. The cortical perimeter was 104.2 ± 4.04 and 103.7 ± 10.35 μm, and the cortical thickness was 6.76 ± 1.07 and 6.49 ± 0.99 mm for the control and T2DM groups, respectively.

**Fig. 2:**
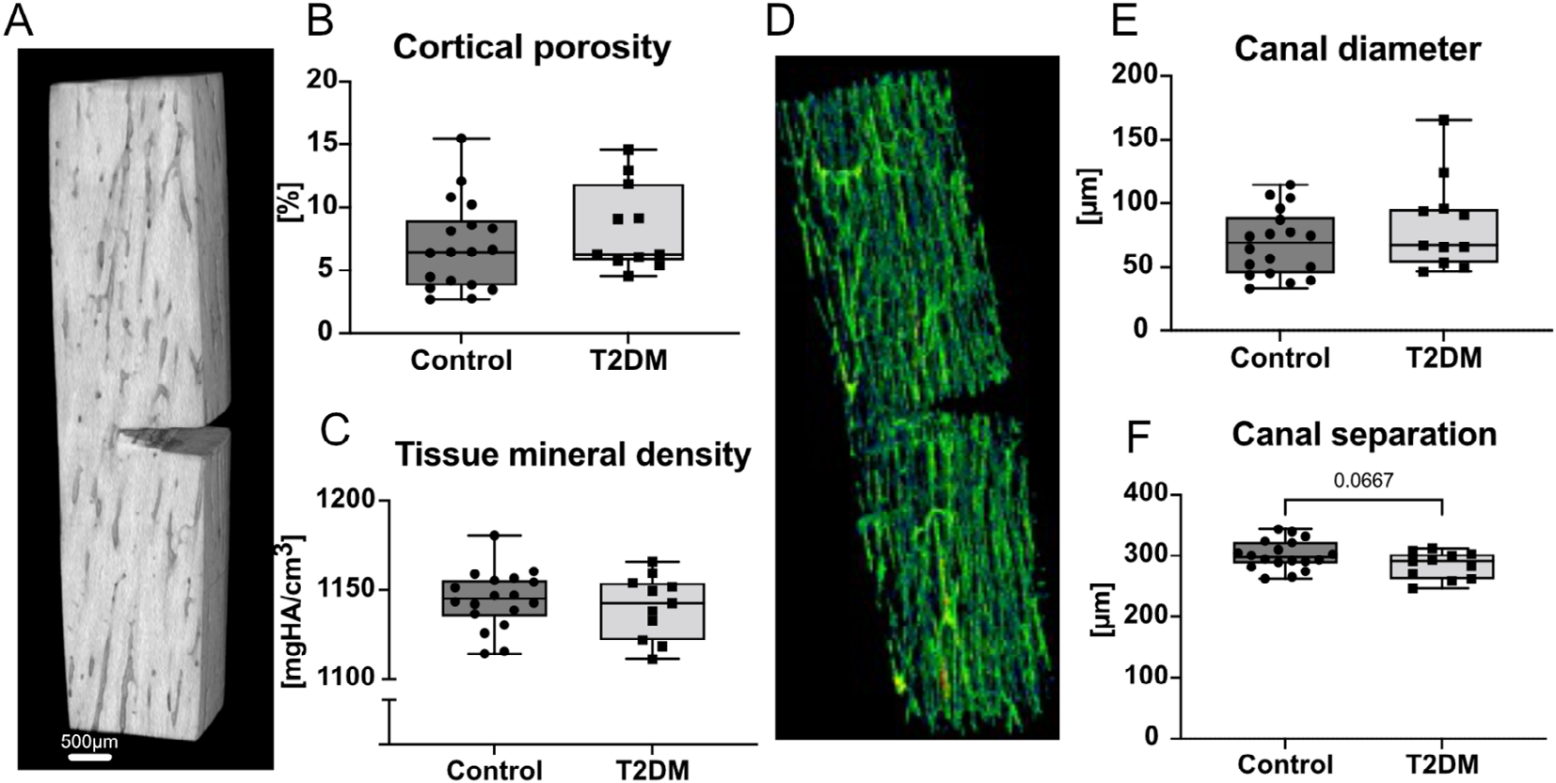
Bone sample microstructural analysis of control and T2DM cases. (*A*) 3D reconstruction of micro–CT scanned bone sample. (*B*) Cortical porosity and (*C*) tissue mineral density do not significantly differ between both groups. (*D*) 3D reconstruction of canal structure and canal indices, (*E*) canal diameter, and (*F*) canal separation.

Bone microstructure was further analyzed using μCT. Fig. 2*A* shows the reconstructed 3D image of a representative cortical bone sample after three‐point bend testing scanned at a resolution of 6 μm. Cortical porosity and tissue mineral density did not differ significantly between both groups (Fig. 2*B,C*). This was further confirmed by the analysis of canal parameters (Fig. 2*D*), showing no significant differences in canal diameter (Fig. 2*E*) but a tendency (*p* = 0.0667) to lower canal separation in the T2DM group (Fig. 2*F*).

**Fig. 3:**
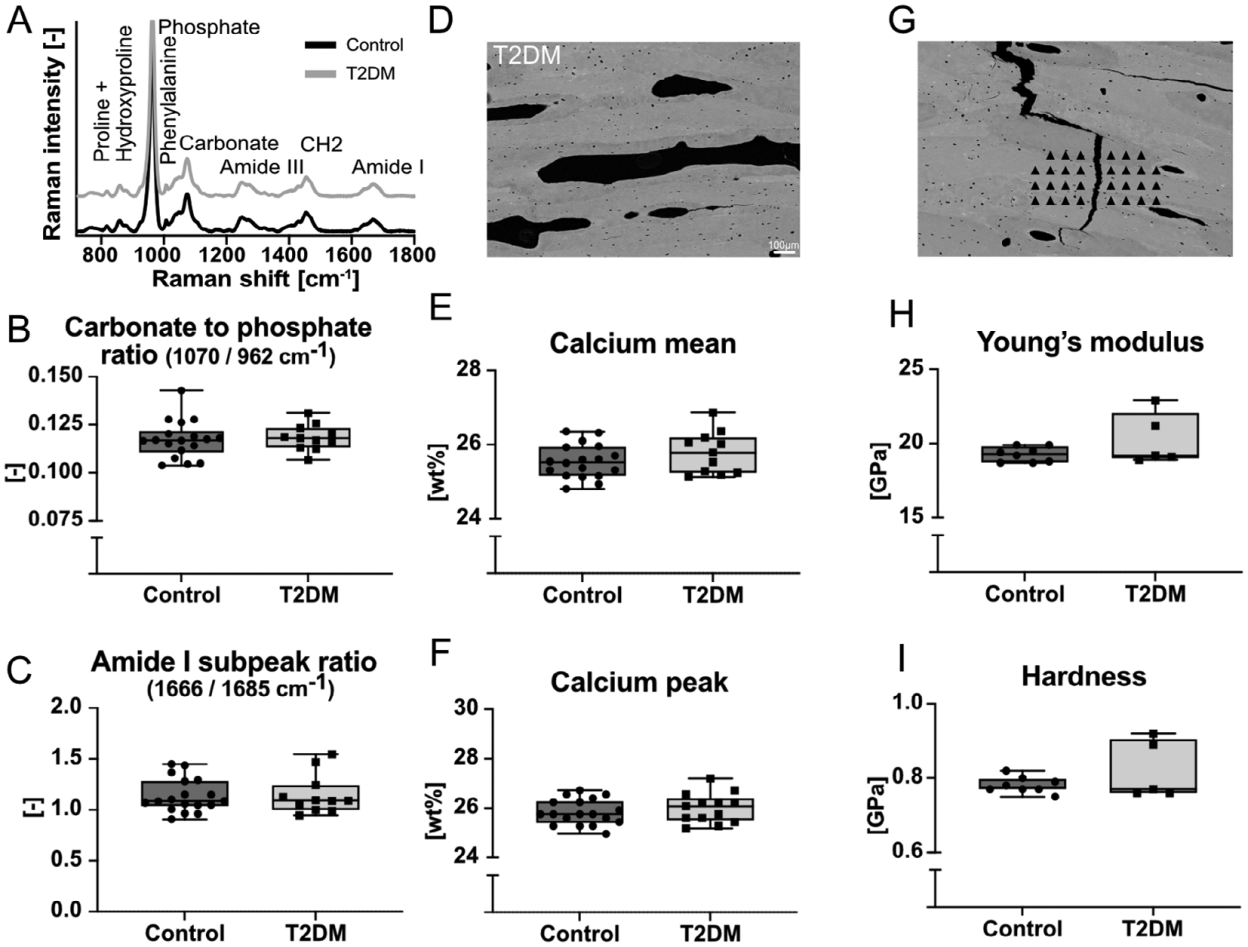
Bone quality analyses of control and T2DM cases combining Raman spectroscopy, quantitative back‐scattered electron imaging (qBEI), and nanoindentation. (*A*) Averaged Raman spectra of both groups. (*B*) Carbonate‐to‐phosphate ratio and (*C*) collagen subpeak ratios indicate no significant differences in bone matrix composition. (*D*) Representative image obtained with qBEI, (*E*) average calcium weight percentage, and (*F*) highest calcium weight percentage show no significant differences in mineralization profiles around crack path. (*G*) Nanoindentation was performed in vicinity of crack path. (*H*) Young's modulus and (*I*) hardness show no significantly different values for a subset of samples from both groups.

To determine bone material quality, we combined several approaches. First, we performed Raman spectroscopy on the unembedded hydrated bone sample (Fig. 3*A*), indicating no significant differences in bone matrix composition in both groups, shown by a carbonate‐to‐phosphate ratio of 0.1173 ± 0.0097 in the control group and 0.1184 ± 0.0067 in the T2DM group and no significant differences in collagen quality of 1.137 ± 0.163 and 1.149 ± 0.194 in the control and T2DM groups, respectively. Similarly, other parameters showed no significant differences (Table S1). For a detailed look at the mineralization degree of the bone tissue around the crack path, we applied qBEI, which is based on calcium weight percentages. The average calcium content was 25.54 ± 0.45 and 25.79 ± 0.57 calcium weight percentage, while the most frequent calcium weight percentage measured was 25.84 ± 0.51 and 26.01 ± 0.6 calcium weight percentage for control and T2DM groups, respectively. Data on the mineralization heterogeneity showed no significant differences and can be seen in Table S1. On a smaller sample set, nanoindentation was performed in the vicinity of the crack path. This reflected the results seen in the mineralization parameters, where there were no significant differences in Young's modulus (control: 19.26 ± 0.5 GPa and T2DM: 20.26 ± 1.74 GPa) and hardness (control: 0.78 ± 0.02 GPa and T2DM: 0.82 ± 0.08 GPa).

**Fig. 4:**
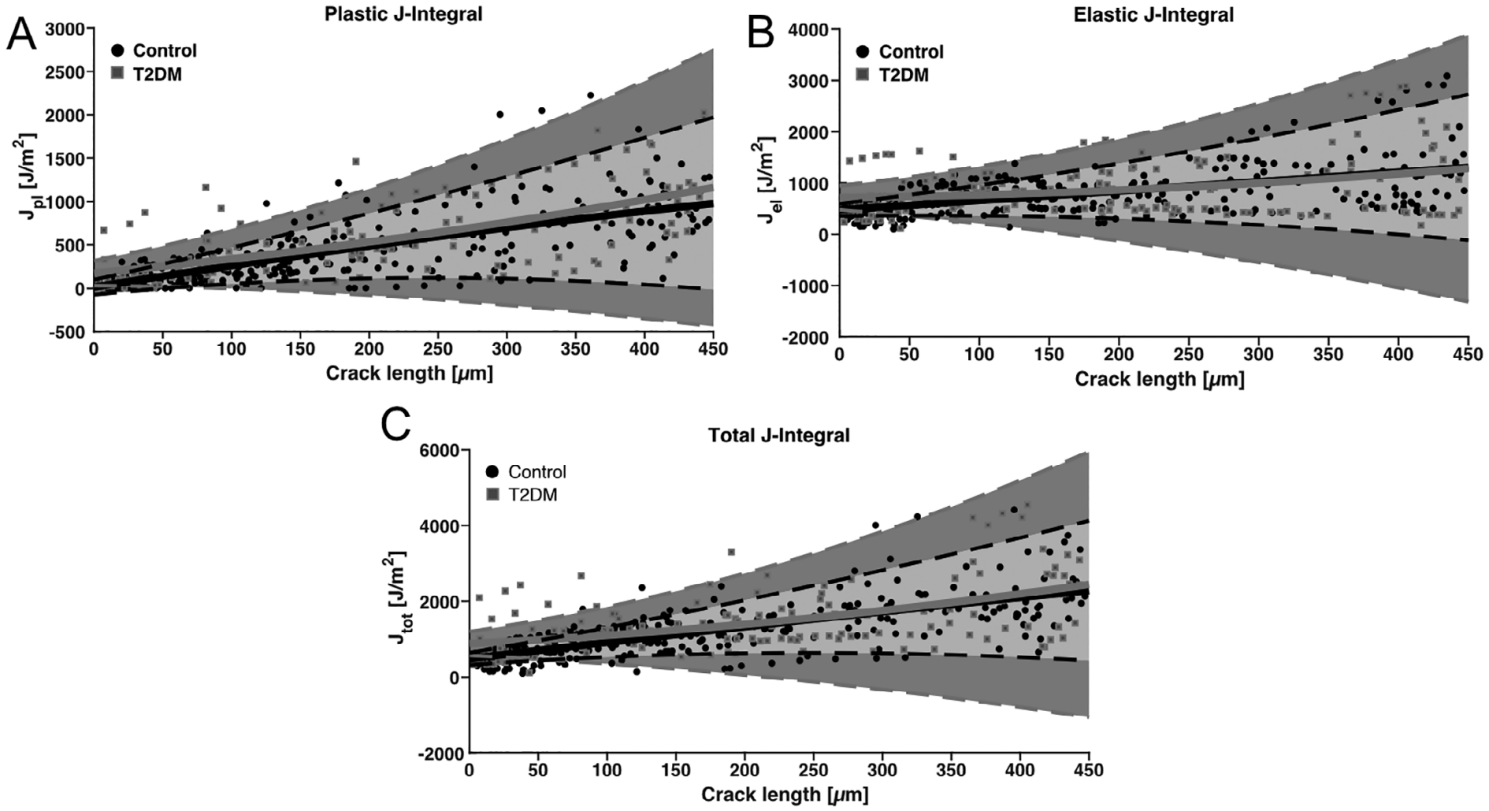
Three‐point bending tests of cortical bone samples from T2DM and healthy controls. (*A*) Plastic J‐integral curves, (*B*) elastic J‐integral curves, and (*C*) total J‐integral curves, all showing individual points of each sample and second‐order fitted curves for control (black) and T2DM (gray) groups including 95% confidence interval.

Using a three‐point bending test, the bone samples were loaded, and crack propagation was visualized with a brightfield microscope. Based on the load–displacement curves and crack propagation measurements, the plastic, elastic, and total J‐integrals were determined. Fig. 4 displays the individual data points of all samples and second‐order fitting lines for control and T2DM samples. While the T2DM fitting line is slightly above the control line, no difference is observed for plastic J‐integral (Fig. 4*A*), elastic J‐integral (Fig. 4*B*), and total J‐integral (Fig. 4*C*).

## Discussion

In this study, we analyzed human cortical bone from male individuals with T2DM compared to age‐matched healthy controls in a case‐controlled post mortem study to assess bone material quality in combination with fracture toughness. While compression testing of trabecular bone from T2DM patients was previously performed,^(^ ^9^, ^10^, ^11^ ^)^ the toughness properties of cortical diabetic bone have rarely been studied. In our cohort of bone samples with a nonsignificantly different microstructure, shown by the analysis of bone and canal structures using μCT, we saw no significant differences in bone matrix composition, mineralization profile around the crack path, and biomechanical properties at the nanolength scale. These similarities were also apparent in fracture toughness behavior and failure characteristics, which did not significantly differ between the groups.

While we could not identify differences in bone material quality indices in this cohort, we and others showed differences in the mineral and collagen properties of bone tissue from individuals with T2DM. Previous studies were performed on a variety of different tissues, such as femoral head, femoral neck, or cortical bone from mid‐diaphyseal femur and tibia, with a focus on different compartments, i.e., trabecular and cortical bone. When studying human bone tissue, high variation is often observed, which can be augmented through the complexity of T2DM, which poses an additional challenge in the identification of a clear underlying mechanism contributing to the pathophysiology of diabetic bone fragility at the tissue level. One phenomenon seen in some but not all T2DM postmenopausal women is the presence of high cortical porosity,^(^ ^6^, ^7^ ^)^ which was shown to be accompanied by compositional changes, as studied previously by our group in a cohort of both male and female individuals.^(^ ^4^, ^5^ ^)^ In our previous study of male individuals with no significant difference in cortical porosity, we identified differences in collagen fibril deformation in T2DM individuals^(^ ^8^ ^)^ by tensile testing with simultaneous synchrotron X‐ray diffraction to measure the deformation of collagen fibrils, i.e., a structure at the nanolength scale of bone tissue. It is possible that changes at this length scale are compensated at larger length scales, which can lead to the results presented here.

Furthermore, sex‐specific differences should be taken into account when assessing and discussing fracture risk and bone material quality indices in T2DM individuals. Currently, more men live with diabetes mellitus than women,^(^ ^28^ ^)^ and men are younger and have a lower BMI when diagnosed.^(^ ^29^ ^)^ Nonetheless, at the time of diagnosis, women bear a greater burden of risk factors and face a higher relative risk of cardiovascular disease. Conversely, in men, a higher absolute risk exists, where cardiovascular events remain more prevalent.^(^ ^29^ ^)^ Hormone levels play a significant role in the risk of developing T2DM, with women having a higher risk when they have elevated testosterone levels, while men face an increased risk when their testosterone levels are low.^(^ ^29^ ^)^ Postmenopausal women are at a higher risk of osteoporosis and fragility fractures than men due to the decline in estrogen levels. Additionally, individuals with T2DM who have microvascular disease exhibit cortical bone deficits.^(^ ^30^ ^)^ Hence, to specifically investigate bone material changes in T2DM and account for sex‐related influences on our outcomes, we have chosen to exclusively study male individuals, as we did in our previous study^(^ ^8^ ^)^ and was done by others.^(^ ^11^ ^)^ Further research is warranted to elucidate the sex‐specific variations in the pathophysiology of bone fragility associated with T2DM to support the development of tailored diagnostic and treatment approaches for both men and women.

Previous post mortem analysis of human cortical bone tissue of different aged donors using three‐point bending test showed lower crack initiation toughness and crack growth toughness with age, including one aged sample with diabetes mellitus, which presented with lower growth toughness and initiation toughness compared to bone from young donors, not to bone tissue from healthy aged donors.^(^ ^31^ ^)^ Lower fracture toughness with age in human cortical bone has also been shown by others.^(^ ^14^, ^15^ ^)^ Additionally, lower fracture toughness properties with age were confirmed, while collagen network integrity best explained variances in fracture toughness.^(^ ^16^, ^17^ ^)^ While these two studies included three individuals with diabetes mellitus, the former study did not aim at determining fracture toughness in diabetic individuals, and the low number of diabetic cases did not allow for drawing conclusions on fracture toughness behavior within the cohort.

Ribosylation is often performed on animal or cadaveric human bone tissue as an artificially induced glycosylation of the bone matrix to simulate diabetes‐related bone matrix changes and subsequent study bone fracture toughness. Ribosylation of bovine bone for 40 days resulted in improved fracture toughness properties such as higher critical fracture toughness and higher J_el_, while J_pl_ did not significantly differ compared to control samples.^(^ ^32^ ^)^ Incubation of bovine tibial bone for 15 days resulted in lower J_pl_ when tested in quasi‐static condition (10^−3^ mm/s) but no differences in J‐integral, either elastic or plastic, were observed when tested in fall‐like conditions (10 mm/s).^(^ ^33^ ^)^ These data indicate that incubation time and testing strain rate significantly influence the results obtained using ex vivo ribosylation. This points to a major influence of disease duration on bone fracture susceptibility. Due to the post mortem nature of our study, diabetes duration was not accessible in the autopsy protocols. A Swedish cohort study suggested that T2DM may not per se induce an increased fracture risk but that specific risk factors, such as diabetes disease duration, tremendously increase fracture risk in the T2DM population.^(^ ^34^ ^)^ While this might not affect every T2DM patient, the authors postulate that 21 million individuals are affected by a risk factor profile contributing to increased fracture risk.^(^ ^34^ ^)^ A longer diabetes duration signifies prolonged high glucose condition within the body, which affects osseos cellular activity, leading to low bone turnover in T2D, which can result in more highly mineralized bone tissue and absence of microcrack removal. Additionally, the longer the high glucose condition continues, the more AGEs can be formed and, thus, impair collagen properties within the bone tissue. Unknown disease duration and differences between individuals may explain why no statistically significant differences were found in this study. However, the presented study design allowed us to obtain sufficiently large bone samples for three‐point bending tests and to control for the orientation of microstructural features that can impact the mechanical properties of bone tissue.

Another approach to determining fracture toughness in diabetes conditions are diabetic small animal models. In Zucker Diabetic‐Sprague Dawley rats, a higher resistance to crack initiation was observed at 16 and 22 weeks of age compared to control rats, with a decline in toughness with aging and, thus, no difference at 29 weeks of age.^(^ ^35^ ^)^ In diabetic TallyHo mice, a lower toughness was observed at 16 and 34 weeks of age, which did not become worse with age/disease duration, while initiation toughness and cracking toughness were the same compared to control mice.^(^ ^36^ ^)^ In a high‐fat‐diet‐induced obesity mouse model, whole femoral fracture toughness was reduced along with larger bone size but less ordered osteocytes and lamellar structure and poorer mineral organization, suggesting that bone material properties may influence size‐independent mechanical properties.^(^ ^37^ ^)^ Lower initiation toughness and lower maximum toughness in combination with higher AGE accumulation was seen in high‐fat‐diet mice compared to low‐fat‐diet mice at 32 weeks of age.^(^ ^38^ ^)^ These data suggest an overall lower fracture toughness in bone from small diabetic animals. However, these models are often not representative of diabetic bone changes seen in humans, where either none or cortical rather than trabecular bone microstructural changes are observed. Furthermore, the bone specimens tested are very different, as human cortical bone is prepared in rectangle‐shaped samples, while in small‐animal studies whole femora are notched and tested, resulting in a cylindrical sample geometry.

This study has a few limitations. We used a three‐point bending setup under environmental conditions, capturing changes in crack propagation at the surface of the bone samples using an optical microscope, while keeping the bone sample hydrated with PBS droplets. While this approach avoided the dehydration of the samples and, as such, a reduction in crack initiation toughness and crack growth resistance, as shown by others,^(^ ^39^ ^)^ visualization of crack propagation in our study was limited to the bone surface and captured manually at varying time points. Continuous automatic recording of the bone surface rather than manual capture would improve crack propagation measurement. Furthermore, the used machine was generated to control for stable crack growth in less heterogeneous material than bone tissue. Therefore, temporary partial deloading occurred when the slope of the force–displacement curve was below a certain threshold. These unloading phases were not always synchronized with crack growth at the bone surface. This can be understood because crack growth can also happen only in the inner part of the sample and, thus, is not visible on the surface. However, this is a principal limitation of optical crack length determination. With a larger support span of 40 mm and two position encoders at the specimen, the average crack length can be precisely determined with the compliance method. Unfortunately, up to now this has not been possible with a support distance of 10 mm due to technical reasons. We agree with others researchers^(^ ^13^ ^)^ that more standardized procedures for cortical bone crack growth determination are needed to improve fracture toughness testing and comparison between individual studies. Here, we applied a variety of techniques (ex vivo clinical imaging, μCT, spectroscopy, electron microscopy, and nanoindentation) to characterize bone material quality. While this allowed us to assess bone material quality at several length scales and different properties, additional indices, such as water content or individual advanced glycation end products (e.g., carboxymethyl‐lysine or pentosidine), have not been addressed in this study but can influence the fracture properties of diabetic bone and need to be taken into consideration in future work.

In conclusion, our study showed that cortical bone from T2DM individuals with no significant differences in microstructure and bone material quality as compared to age‐matched healthy individuals also showed no significant differences in fracture toughness. These data support recent studies demonstrating that not all T2DM patients but measurable subgroups of patients experience an increased fracture risk potentially due to a specific risk profile. To what extent bone matrix and fracture toughness properties within these patient subgroups differ remains to be elucidated in the future.

## Author Contributions

**Eva M. Wölfel:** Conceptualization; data curation; formal analysis; funding acquisition; investigation; methodology; supervision; visualization; writing – original draft; writing – review and editing. **Benjamin Bartsch:** Data curation; formal analysis; investigation. **Jasmin Koldehoff:** Conceptualization; data curation; funding acquisition; investigation; methodology. **Imke A. K. Fiedler:** Formal analysis; investigation; methodology; resources; supervision; writing – original draft; writing – review and editing. **Sofie Dragoun‐Kolibova:** Investigation; methodology. **Felix N. Schmidt:** Formal analysis; investigation; methodology. **Johannes Krug:** Formal analysis; investigation; methodology; software; visualization; writing – original draft; writing – review and editing. **Mei‐Chun Lin:** Conceptualization; data curation; formal analysis; investigation; visualization; writing – original draft; writing – review and editing. **Klaus Püschel:** Investigation; resources. **Benjamin Ondruschka:** Investigation; resources; writing – review and editing. **Elizabeth A. Zimmermann:** Formal analysis; resources; validation. **Hans Jelitto:** Formal analysis; investigation; methodology; resources. **Gerold Schneider:** Investigation; methodology; resources; validation. **Bernd Gludovatz:** Formal analysis; investigation; methodology; validation; writing – review and editing. **Björn Busse:** Conceptualization; formal analysis; funding acquisition; methodology; project administration; resources; writing – original draft; writing – review and editing.

## Disclosures

No authors have conflicts of interest to disclose.

### Peer Review

The peer review history for this article is available at https://www.webofscience.com/api/gateway/wos/peer‐review/10.1002/jbm4.10839.

## Supporting information


**Table S1.** Bone Material Quality Indices Measured with Raman Spectroscopy and Quantitative Backscattered Electron Imaging.

## Data Availability

The datasets generated and/or analyzed for this study are not publicly available but can be obtained from the corresponding author on reasonable request. All data needed to evaluate the conclusion in the paper are presented in the paper.
