# Supplementary material for: When Cortical Bone Matrix Properties Are Indiscernible between Elderly Men with and without Type 2 Diabetes, Fracture Resistance Follows Suit
Source: JBMR Plus. 2023 Dec 1;7(12):e10839. doi: 10.1002/jbm4.10839 (PMC10731113; doi:10.1002/jbm4.10839)
Supplement: Supplementary file 1 — Table S1. Bone Material Quality Indices Measured with Raman Spectroscopy and Quantitative Backscattered Electron Imaging. [file JBM4-7-e10839-s001.docx]

Supplementary Table 1. Bone Material Quality Indices Measured with Raman Spectroscopy and Quantitative Backscattered Electron Imaging

|  | **Parameter** | **Control** | **T2DM** |
| --- | --- | --- | --- |
| Raman spectroscopy | Mineral to matrix ratio:  v1 phosphate / amide I  (962 cm^-1^/ (1600-1720 cm^-1^)) | 4.709 ± 0.559 | 4.469 ± 0.535 |
|  | Mineral to matrix ratio:  v1 phosphate / amide III  (962 cm^-1^/ (1215-1365 cm^-1^)) | 3.945 ± 0.256 | 3.902 ± 0.262 |
|  | Carbonate to amide I ratio  (1070 cm^-1^/1665 cm^-1^) | 0.5498 ± 0.0563 | 0.5279 ± 0.0593 |
|  | Crystallinity  (1/FWHM of v1 phosphate 962 cm^-1^) | 0.0621 ± 0.001 | 0.0625 ± 0.0009 |
| qBEI | Calcium heterogeneity [wt %] | 2.097 ± 0.139 | 2.145 ± 0.082 |

Mineral to matrix ratios, carbonate to amide I ratio and crystallinity measured with Raman spectroscopy do not differ in a cohort of male individuals with and without type 2 diabetes mellitus. Heterogeneity of calcium weight assessed with quantitative backscattered electron imaging showed no significant difference between the two groups.

T2DM = type 2 diabetes mellitus; qBEI = quantitative backscattered electron imaging.
